# Use of nonsteroidal anti-inflammatory drugs in corneal epithelial ingrowth due to traumatic flap dislocation after LASIK: Case report

**DOI:** 10.1097/MD.0000000000037333

**Published:** 2024-03-01

**Authors:** Mengman Gao, Zhong Shi, Xiujin Guo

**Affiliations:** a Department of Ophthalmology, The Second Hospital of Hebei Medical University, Shijiazhuang, China.

**Keywords:** case report, epithelial ingrowth, eye trauma, laser in situ keratomileusis, nonsteroidal anti-inflammatory drugs

## Abstract

**Rationale:**

Ophthalmologists mainly treat epithelial ingrowth by lifting the flap and scraping the ingrowth or using scraping combined with phototherapeutic keratectomy, mitomycin C, and so on. The potential usefulness of nonsteroidal anti-inflammatory drugs in such circumstances has not been reported before.

**Patient concerns:**

A 32-year-old man and a 25-year-old man underwent lifting and scraping of the flap and phototherapeutic keratectomy to remove the epithelial ingrowths. Unfortunately, the ingrowths recurred and continued to develop.

**Diagnosis:**

The patients were diagnosed with corneal epithelial ingrowth.

**Interventions:**

The administration of bromfenac sodium and fluorometholone eye drops.

**Outcomes:**

Epithelial ingrowths in both patients disappeared after 6 and 1 month of treatment, respectively. There were no adverse reactions to the eye drops.

**Lessons:**

Nonsteroidal anti-inflammatory drugs may be broadly applied in the treatment of epithelial ingrowth after laser in situ keratomileusis.

## 1. Introduction

Laser in situ keratomileusis (LASIK) is one of the primary types of refractive surgery. However, epithelial ingrowth due to traumatic flap dislocation is a serious complication that may occur after LASIK.^[1,2]^ Ophthalmologists mainly treat this complication by lifting the flap and scraping the ingrowth or using scraping combined with phototherapeutic keratectomy (PTK),^[3]^ mitomycin C,^[4]^ neodymium-doped yttrium-aluminum-garnet laser,^[5]^ and other treatments. This report presents 2 patients who developed post-LASIK epithelial ingrowth due to trauma and discusses the effect of nonsteroidal anti-inflammatory drugs (NSAIDs) on the inhibition of corneal epithelial cell proliferation. The potential usefulness of NSAIDs in such circumstances has not been reported before.

## 2. Case reports

### 2.1. Case 1

**Figure 1.**
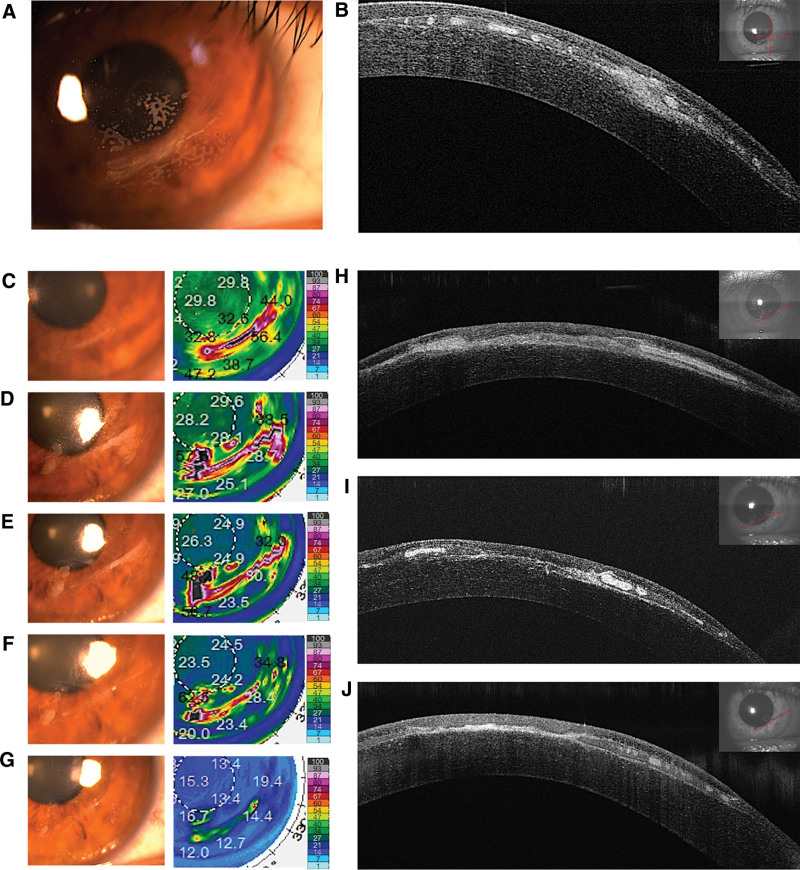
Slit-lamp photographs, Pentacam densitometry, densitometry map color-scale images and optical coherence tomographic images of Case 1. (A, B) The cornea with epithelial ingrowth caused by trauma 2 mopreviously. (C) One week after combined lifting and scraping with phototherapeutic keratectomy (PTK). (D, H) One mo after combined lifting and scraping with PTK. (E, I) One mo after treatment with bromfenac sodium (0.1%) and fluorometholone (0.1%). (F) Two mo and (G, J) 3 mo after treatment.

A 32-year-old man presented to the Department of Ophthalmology, Excimer Refractive Center, Second Hospital of Hebei Medical University on August 19, 2019, with the chief complaint of cloudy vision in his right eye. The patient underwent a first LASIK surgery to correct myopia 14 years ago. On admission day, slit-lamp examination revealed an arc-shaped stromal scar surrounded by a map-like epithelial ingrowth (Fig. 1A). Anterior segment optical coherence tomography (AS-OCT) (RetinaScan-3000; NIDEK, Gamagori, Japan) showed multifocal epithelial nests beneath the corneal flap (Fig. 1B). Therefore, the patient underwent corneal epithelial lifting and scraping combined with PTK (Fig. 1C).

One month after the surgery, the epithelial ingrowth was larger and thicker than before (Fig. 1D and H) After almost 1 month of treatment with bromfenac sodium hydrate ophthalmic solution and fluorometholone eye drops, the epithelial ingrowth lesion had decreased to a size smaller than that before treatment (Fig. 1E–G). Moreover, AS-OCT examination showed thinning of the epithelial ingrowth (Fig. 1I–J). There were no adverse reactions or irritation during the medication.

### 2.2. Case 2

**Figure 2.**
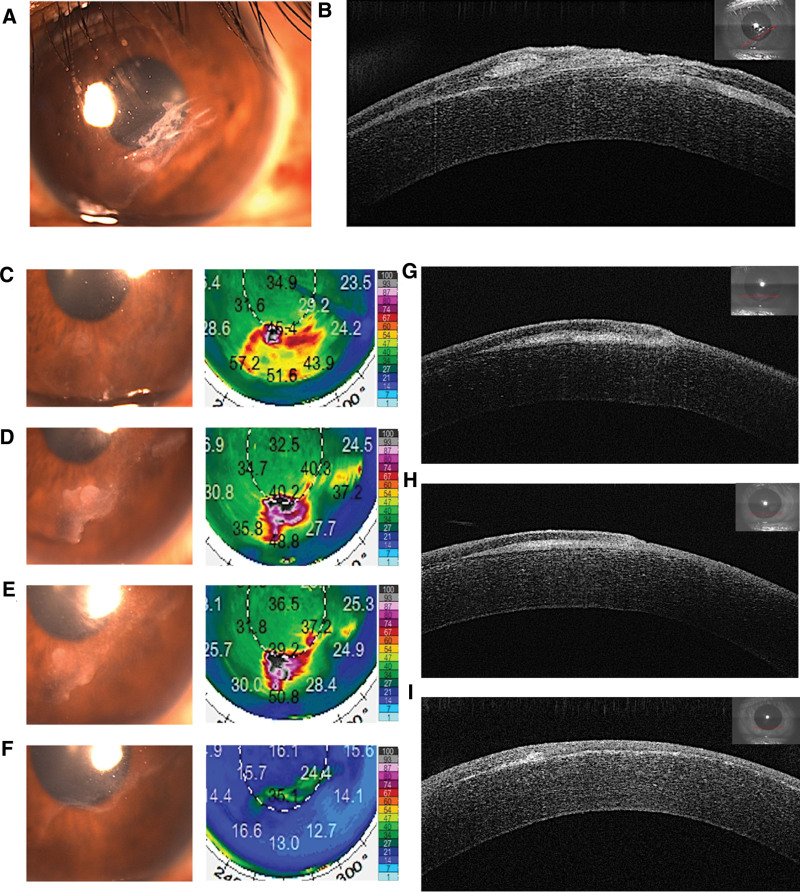
Slit-lamp photographs, Pentacam densitometry, densitometry map color-scale images and optical coherence tomographic images of Case 2. (A, B) Two mo after trauma. (C) One wk after combined lifting and scraping with phototherapeutic keratectomy (PTK). (D, G) Two wk after combined lifting and scraping with PTK was performed. (E, H) One wk after treatment with bromfenac sodium (0.1%) and fluorometholone (0.1%). (F, I) Two mo after treatment with the drugs.

A 25-year-old man who underwent LASIK surgery 2 years ago presented to the Second Hospital of Hebei Medical University on December 16, 2019. The patient could not see clearly for about 1 month. The examination and scan results showed cascade folding, large areas of implanted corneal epithelium, and a dissolved portion of the corneal flap (Fig. 2A–B). One day after admission, the patient underwent left eye flap curettage plus PTK (Fig. 2C)..

Fifteen days after the surgery, AS-OCT examination showed a recurrence of the epithelial ingrowth (Fig. 2D and G). After a week of using bromfenac sodium hydrate ophthalmic solution and fluorometholone eye drops, the patient symptoms disappeared (Fig. 2E and H). At 3 months postoperatively, Slit-lamp examination showed no active epithelial ingrowth (Fig. 2F and I). There were no adverse reactions or irritation during the medication.

## 3. Discussion

This report aimed to present the details of 2 cases of epithelial ingrowth after LASIK and traumatic flap dislocation and their management using NSAIDs. The treatment was successful in both cases.

The most common cause of epithelial ingrowth after LASIK is trauma. Stromal adhesion in the cornea after LASIK comprises approximately 25% to 50% of a normal cornea. This makes the cornea vulnerable to trauma-induced flap displacement. For both cases in this report, the epithelial ingrowth recurred despite surgical interventions. Corneal slit-lamp examination results at each follow-up visit showed that the initial epithelial ingrowth was more concentrated on the corneal flap or stromal scar than at other sites. Based on the authors’ experience, it could be hypothesized that some corneal epithelial cells might have remained in the interstitial space of the stroma or corneal flap laceration when the epithelium was scraped during scraping and PTK. Because the corneal flap had partially dissolved and thinned preoperatively in both cases, LASIK retreatment would have increased the risk of epithelial implantation.

Topical steroids are often used to treat epithelial ingrowths. In Case 1, the patient was treated with a tobramycin/dexamethasone combination eye drops for 1 month postoperatively. During this period, the epithelial ingrowth area gradually expanded and thickened. It was speculated that the patient was not responsive to topical steroids. Wu et al^[6]^ have previously discussed the methods to inhibit corneal epithelial cells in vitro using diclofenac sodium and showed that diclofenac sodium could induce corneal epithelial cell apoptosis. NSAIDs are also believed to inhibit the conversion of arachidonic acid to prostaglandin by inhibiting cyclooxygenase activity, thereby affecting DNA and protein synthesis in the epithelial cells.^[6]^ Compared with diclofenac sodium, the bromine in bromfenac sodium can improve its lipophilicity and promote its passage through the tissues and cell membrane, thus extending the duration of its action.^[7]^ Bromfenac sodium also has a greater inhibitory effect on cyclooxygenase-2 than diclofenac sodium.^[8]^ Therefore, we instructed the patient to discontinue tobramycin and dexamethasone administration, and prescribed bromfenac sodium eyedrops combined with fluorometholone eye drops. For both Cases 1 and 2, the area of epithelial ingrowth did not expand further and almost disappeared after 7 and 1 months, respectively. Still, further experimental studies are needed to confirm this conclusion.

For both cases in this report, slit-lamp biomicroscopy, Pentacam imaging, and AS-OCT scans were used to track the changes in the patients’ conditions, as these modalities can provide a more comprehensive evaluation of the cornea. Corneal density measurement can be used as an objective index to observe epithelial ingrowth.^[9]^ AS-OCT can more accurately assess the degree of epithelial ingrowth.^[10]^ The size of the gray-scale area of corneal density had increased before NSAIDs administration, the epithelial ingrowth had thickened, and the area of corneal epithelial ingrowth had increased. After treatment, the lesion became thinner and smaller. The epithelial bundle under the flap was relatively thin and dissipated faster when closer to the flap edge. Therefore, it was speculated that the duration of corneal epithelial dissipation was associated with the area, position, and thickness of the corneal epithelium.

To the best of the authors’ knowledge, this is the first report to describe the effects of bromfenac sodium hydrate ophthalmic solution on epithelial ingrowth. For refractory corneal epithelial implantation after LASIK and traumatic flap dislocation, conservative treatment with bromfenac sodium might be considered. It appears safe, effective, and less expensive than repeated surgeries to eliminate corneal epithelial implants. This regimen might eliminate the risk of re-epithelialization and other related complications associated with repeated surgeries.

## Author contributions

**Conceptualization:** Mengman Gao.

**Data curation:** Mengman Gao, Zhong Shi, Xiujin Guo.

**Formal analysis:** Mengman Gao, Zhong Shi, Xiujin Guo.

**Project administration:** Mengman Gao.

**Writing – original draft:** Mengman Gao, Zhong Shi, Xiujin Guo.

**Writing – review & editing:** Mengman Gao, Zhong Shi, Xiujin Guo.
